# Structure-Guided Discovery of Benzoic-Acid-Based TRPC6 Ligands: An Integrated Docking, MD, and MM-GBSA SAR Study: Potential Therapeutic Molecules for Autism Spectrum Disorder

**DOI:** 10.3390/ph18101577

**Published:** 2025-10-18

**Authors:** Nicolás Ignacio Silva, Gianfranco Sabadini, David Cabezas, Cristofer González, Paulina González, Jiao Luo, Cristian O. Salas, Marco Mellado, Marcos Lorca, Javier Romero-Parra, Jaime Mella

**Affiliations:** 1 Departamento de Química Orgánica, Facultad de Química y de Farmacia, Pontificia Universidad Católica de Chile, Santiago de Chile 7820436, Chile; nsilvas@estudiante.uc.cl (N.I.S.); cosalas@uc.cl (C.O.S.); 2 Instituto de Química, Facultad de Ciencias, Universidad de Valparaíso, Av. Gran Bretaña 1111, Valparaíso 2360102, Chile; gianfranco.sabadini@postgrado.uv.cl (G.S.); cristofer.gonzalez@postgrado.uv.cl (C.G.); paulina.gonzalezd@postgrado.uv.cl (P.G.); jiao.luo@postgrado.uv.cl (J.L.); 3 Departamento de Ciencias Biológicas y Químicas, Facultad de Ciencias, Universidad San Sebastián, Campus Los Leones, Lota 2465, Providencia, Santiago 7510157, Chile; dcabezasg@docente.uss.cl; 4 Centro de Investigación en Ingeniería de Materiales, Universidad Central de Chile, Santiago 8330507, Chile; marco.mellado@ucentral.cl; 5 Facultad de Ciencias de la Vida, Carrera de Química y Farmacia, Universidad Viña del Mar, Viña del Mar 2520000, Chile; marcos.lorca@uvm.cl; 6 Organic Chemistry and Physical Chemistry Department, Faculty of Chemical and Pharmaceutical Sciences, Universidad de Chile, Olivos 1007, Santiago 7820436, Chile; 7 Centro de Investigación, Desarrollo e Innovación de Productos Bioactivos (CInBIO), Universidad de Valparaíso, Av. Gran Bretaña 1111, Valparaíso 2360102, Chile

**Keywords:** autism spectrum disorder, TRPC6, benzoic acids, benzothiophene, agonists, molecular dynamics

## Abstract

**Background**: TRPC6 is recognized as a therapeutically relevant cation channel, whose activation is governed by specific ligand–pocket interactions. **Methods**: An integrated in silico workflow was employed, comprising structure-based docking, 100-nanosecond molecular dynamics (MD) simulations, and MM-GBSA calculations. Benzoic-acid–based compounds were designed and prioritized for binding to the TRPC6 pocket, using a known literature agonist as a reference for benchmarking. **Results**: Within the compound series, BT11 was found to exhibit a representative interaction profile, characterized by a key hydrogen bond with Trp680 (~64% occupancy), persistent salt-bridge interactions with Lys676 and Lys698, and π–π stacking with Phe675 and Phe679. A favorable docking score (−11.45 kcal/mol) was obtained for BT11, along with a lower complex RMSD during MD simulations (0.6–4.8 Å), compared with the reference compound (0.8–7.2 Å). A reduction in solvent-accessible surface area (SASA) after ~60 ns was also observed, suggesting decreased water penetration. The most favorable binding energy was predicted for BT11 by MM-GBSA (−67.72 kcal/mol), while SOH95 also ranked highly and slightly outperformed the reference. **Conclusions**: These convergent computational analyses support the identification of benzoic-acid–derived chemotypes as potential TRPC6 ligands. Testable hypotheses are proposed, along with structure–activity relationship (SAR) guidelines, to inform experimental validation and guide the design of next-generation analogs.

## 1. Introduction

**Figure 1:**
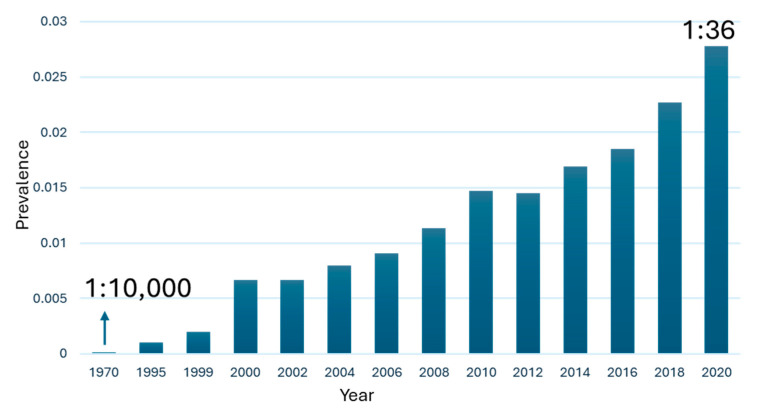
Graph of autism spectrum disorder prevalence over the years, from 1970 to 2020. Between 2000 and 2020, the proportion increased more than fourfold (data obtained from The Autism Community in Action (TACA)).

**Figure 2:**
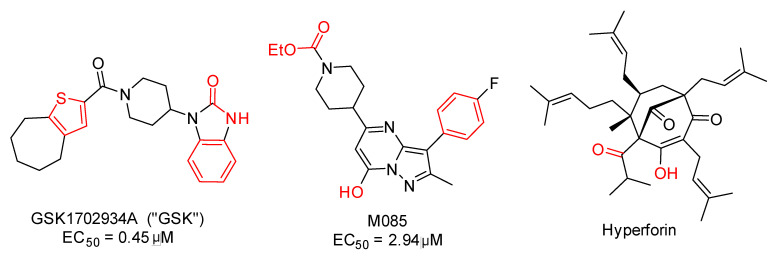
Relevant TRPC6 agonist ligands [29,30]. GSK and M085 are not selective, and they can activate TRCPC3, 6, and 7. Important fragments for the activity are highlighted in red. The EC_50_ (Half maximal effective concentration) value for Hyperforin is not reported. In this study, the GSK compound was used as a standard of comparison, since it is one of the most widely used in comparative studies in the literature, and its interactions with the channel have been extensively studied.

Autism Spectrum Disorder (ASD) is a neurodevelopmental disorder [1,2,3] characterized by persistent challenges in social interaction [4], communication [5], and repetitive patterns of behavior [6]. Approximately 75 million people worldwide have autism spectrum disorder, equivalent to ~1% of the global population [7,8]. The current rate of children with autism is 1 in 36 (Figure 1), and there is no pharmacological cure [9]. Figure 1 summarizes population-based prevalence estimates of autism. Apparent increases across successive cohorts are best explained by enhanced awareness and screening, changes in diagnostic practices (e.g., *Diagnostic and Statistical Manual of Mental Disorders, 5th edition* (DSM-5) criteria consolidation and service-coding incentives), and expanded access to educational/clinical evaluations, rather than by demonstrated growth in the incidence of new cases. Most therapies aim to manage symptoms such as aggression [10], hyperactivity [11], and sleep disorders [12] associated with autism. Among the potential biological targets to treat ASD are metabotropic glutamate receptor 5 (mGluR5) [13,14,15], protein tyrosine phosphatase receptor type D (PTPRD) [16,17], 5-hydroxytryptamine (5-HT) receptors [18,19], γ-aminobutyric acid (GABA) receptors [20,21], and transient receptor potential canonical 6 (TRPC6) [22,23,24,25]. TRPC6 is a member of the transient receptor potential (TRP) channel family, which regulates Ca^2+^, Na^+^, and K^+^ influx in various cells and tissues [26,27,28]. Aberrant TRPC6 channel activity has been associated with neurological disorders, including ASD [22,23,24,25]. TRPC6 is a tetrameric, non-voltage-gated, Ca^2+^-permeable cation channel whose recent cryo-electron microscopy (cryo-EM) structures mapped discrete modulator sites, including an agonist pocket in the extracellular cavity formed by S6 and the pore helix—consistent with direct activation by the native lipid diacylglycerol (DAG) [26]. In neurons, TRPC6 signaling engages Ca^2+^-dependent pathways (e.g., Brain-derived neurotrophic factor (BDNF), Calcium/calmodulin-dependent protein kinase IV (CaMKIV), Protein kinase B (Akt), and cAMP response element–binding protein (CREB)) that govern synaptic development and plasticity [25]. Relevance to ASD is supported by human genetics and cellular models: a de novo balanced translocation disrupting TRPC6 was identified in a non-syndromic ASD individual, and Induced pluripotent stem cells (iPSC)-derived neurons from this case showed reduced Ca^2+^ influx, impaired dendritic complexity and excitatory synapses—phenotypes rescued by hyperforin (a TRPC6 agonist) or Insulin-like growth factor 1 (IGF-1) [22,24]. Complementary in vivo work in Drosophila shows that null mutations in trpγ (the TRPC6 homolog) produce ASD-like behavioral defects—including impaired social interactions, hyperactivity, and altered sleep homeostasis—many of which are attenuated by hyperforin, further supporting TRPC6 as an ASD risk gene [22]. Therefore, modulating TRPC6 channel function presents a potential avenue for therapeutic intervention in ASD. In recent years, several agonists and modulators of the TRPC6 channel have been identified (Figure 2), demonstrating the potential to regulate its activity and restore normal calcium signaling [26,29]. However, there is still a need for the development of novel compounds with improved efficacy and selectivity.

TRPC6-affine ligands tend to be highly lipophilic. In Figure 2, we highlight several common features: the presence of multiple hydrogen-bond acceptors (e.g., O, N, and, in some cases, F) together with at least one hydrogen-bond donor (e.g., OH or NH). For M085, hydrolysis of the ethyl ester could generate a negatively charged carbamate function at physiological pH, which may interact with cationic TRPC6 residues such as Lys676 and Lys698. At least one hydrogen-bond acceptor group appears necessary to interact with the key Trp680 residue. The presence of at least one aromatic ring (such as thiophene, benzene, or the pyrazolopyrimidine system) would be relevant for establishing π-stacking interactions with the abundant aromatic and hydrophobic residues in the TRPC6 agonist pocket. By contrast, hyperforin lacks aromatic rings; moreover, its mechanism of action on TRPC6 has not been clarified and is likely not mediated by direct binding to the agonist cavity.

In order to find new and better molecules (with affinity in the nM range and better selectivity profile), we carried out a virtual screening of 202 molecules from our research group in the S6–pore helix agonist site centered on Trp680. This site is different from the cytosolic S1–S4 pocket described for antagonists [26]. We found that the BZ8 compound exhibited a good fit in the agonist groove of TRPC6 and was able to establish a hydrogen bond with Trp680, a key amino acid in agonism [26], but was unable to establish relevant interactions with other important amino acids such as Lys676 and Asn702. To this end, we measured the distances to these residues and determined that the insertion of an OH and a COOH group was promising. After this change, the scoring value improved substantially. Additionally, we found that switching from benzimidazole to benzothiophene further improved the docking score. In this study, we report the design and in silico evidence of agonistic activity for 3-(2-naphthamido)-4-(benzo[b]thiophen-2-yl)-5-hydroxybenzoic acid (compound BT11) and its derivatives (SOH95, INV29, and T18) as potential agonists of the TRPC6 channel, based on molecular docking and molecular dynamics simulations. Our computational discoveries show that compound BT11 outperforms the reference ligand GSK in protein–interaction profiles, contact residence times, and pore-diameter expansion. Therefore, we postulate BT11 as a potential hit compound for the experimental development of carboxylic acid derivatives as TRPC6 channel ligands with potential applications in ASD.

## 2. Results and Discussion

**Figure 3:**
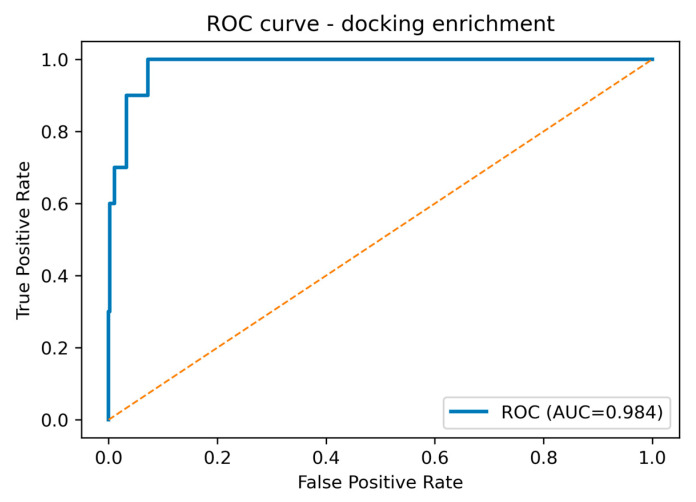
Receiver Operating Characteristic (ROC) curve for the docking enrichment benchmark distinguishing TRPC6 actives from property-matched decoys (*N* = 510; 10 actives, 500 decoys). The solid line shows the empirical ROC (Area under curve (AUC) = 0.984; 95% CI: 0.964–0.998); the dashed diagonal denotes random performance.

The enrichment analysis demonstrates that the docking protocol achieves good discrimination between TRPC6 actives and property-matched decoys. The Area under the Precision–Recall curve (ROC-AUC) of 0.984 (95% Confidence interval (CI): 0.964–0.998) indicates that, across the entire ranked list, an active is almost always scored above a decoy (Figure 3). Importantly, the class imbalance in this benchmark (prevalence 0.02) is appropriately captured by the precision–recall metric: the Area under the Precision–Recall curve (PR-AUC) of 0.703 substantially exceeds the baseline expected under random ranking (=0.02), confirming that high precision can be maintained while recall increases.

Early-recognition performance—most relevant for prospective selection—is similarly strong. A summary of the metrics is reported in Table 1. The top 1% of the list (TopK = 5) contains four true actives, corresponding to Enrichment Factor (EF1)% = 37.36 (95% CI: 18.68–70.05). The top 2% (TopK = 10) contains six actives (EF2% = 28.02; 95% CI: 15.57–42.56), and the top 5% (TopK = 24) contains nine actives (EF5% = 17.51; 95% CI: 9.73–19.46). These values indicate that the protocol concentrates the vast majority of actives at the very top of the ranking, where experimental testing is typically focused, thereby maximizing screening efficiency.

**Figure 4:**
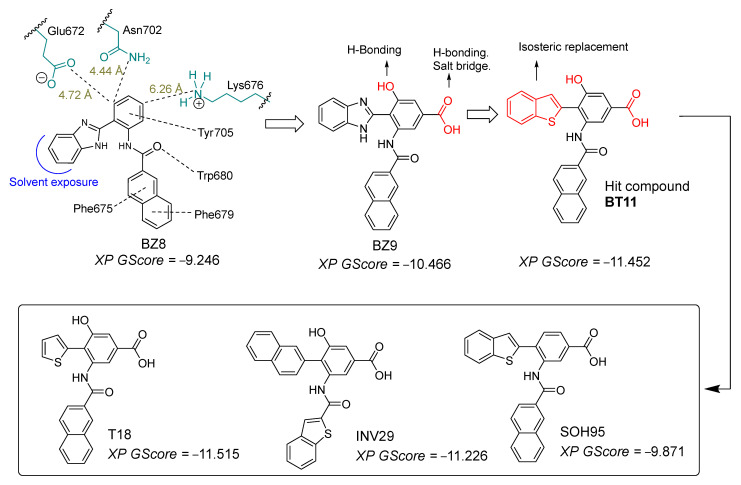
Sequential design strategy of BT11 and its derivatives SOH95, INV29, and T18. The derivative BZ8 exhibited a favorable interaction pattern. To achieve stronger interactions with surrounding residues, an acidic group and a hydroxyl substituent were incorporated. In addition, the benzimidazole moiety was replaced by benzothiophene to prevent the formation of an intramolecular hydrogen bond that hindered interaction with Trp680. The Glide Extra-Precision docking score (XP GScore) is expressed in kcal/mol.

Through a virtual screening of molecules previously synthesized by our research group [31,32,33,34], we identified compound BZ8 as displaying a suitable interaction pattern within the agonist–binding site of TRPC6 (Figure 4). BZ8 established interactions with residues Trp680, Phe675, and Phe679, which are known to be critical for agonist ligand affinity. Additionally, BZ8 formed an interaction with Tyr705. In proximity to the benzene ring of the 2-aryl fragment of the benzimidazole scaffold, residues Glu672 (4.72 Å) and Asn702 (4.44 Å) were located. To promote significant hydrogen-bonding interactions with these residues, we incorporated a hydroxyl (–OH) substituent. Furthermore, the 2-aryl fragment was found to be 6.26 Å from Lys676; therefore, we introduced a carboxylate group (–COO^–^) to enable potential hydrogen bonding and a salt bridge with this residue.

On the other hand, a 100 ns molecular dynamics (MD) simulation of BZ8 revealed that the compound progressively lost the docking-predicted interactions. This behavior was mainly due to the benzimidazole moiety forming a persistent intramolecular hydrogen bond with the amide carbonyl of the 2-naphthyl portion throughout the simulation, thereby preventing effective interaction with Trp680, Phe675, and Phe676. To overcome this limitation, we performed an isosteric replacement of benzimidazole with benzothiophene, which cannot sequester the carbonyl group through intramolecular hydrogen bonding.

In the docking studies, the simple addition of –OH and –COOH groups increased the docking score to −10.466 kcal/mol, while replacement of benzimidazole with benzothiophene further improved the score to −11.452 kcal/mol. The larger size of benzothiophene allows for a different and more favorable accommodation within the binding site compared to benzimidazole.

**Figure 5:**
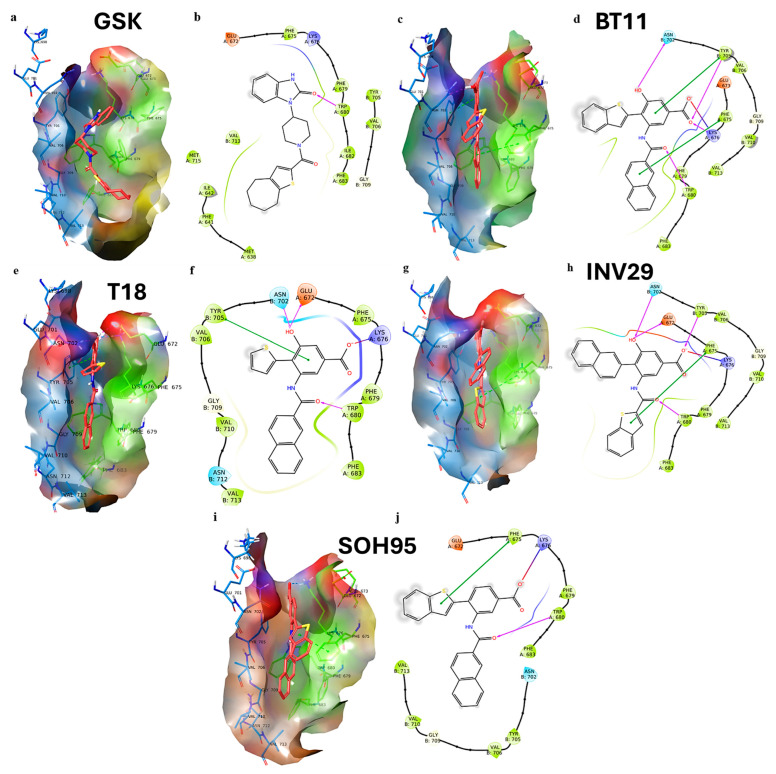
Best 3D and 2D docking poses for GSK (**a**,**b**); BT11 (**c**,**d**); T18 (**e**,**f**); INV29 (**g**,**h**); and SOH95 (**i**,**j**). GSK forms only a single hydrogen bond with Trp680 and is stabilized mainly through hydrophobic interactions. BT11 exhibited the richest interaction pattern within the binding cavity. The 2D interaction maps show residues within 4 Å of the ligands. Hydrophobic amino acids are shown in green, polar residues in light blue, cationic residues in blue, and anionic residues in red.

Starting from the hit compound BT11, we introduced several structural modifications to generate a series of new derivatives: one with a thiophene ring (T18, −11.515 kcal/mol), one with the positions of the naphthalene and benzothiophene groups swapped (INV29, −11.226 kcal/mol), and one lacking the hydroxyl group (SOH95, −9.871 kcal/mol). Figure 5 shows the docking results for this designed series. As a reference, we used the compound GSK, which contains a thiophene ring and an amide function, as well as a benzimidazolone system. This scaffold closely resembles our proposed molecules and provides a suitable basis for structural comparison.

The agonist GSK adopts a curved conformation within the binding cavity (Figure 5a). The cycloheptane–thiophene fragment is oriented toward the hydrophobic portion of the site, whereas the benzimidazolone moiety projects into the more polar region of the cavity. The compound forms a hydrogen bond between the imidazolone carbonyl and Trp680 (1.99 Å), in addition to several hydrophobic contacts with Val713, Met715, and Ile642 (Figure 5b).

For the derivative BT11, the naphthalene and benzoic acid groups are inserted coplanarly within the cavity (Figure 5c), while the benzothiophene fragment adopts an angle of approximately 90° relative to these rings. BT11 forms four hydrogen bonds with Lys676, Trp680, Asn702, and Tyr705 (Figure 5d). The interaction with Trp680 occurs between the amide carbonyl group of the ligand and the NH function of Trp680, acting as a hydrogen bond donor. This interaction is particularly relevant for agonists designed to target TRPC6, and in this case, BT11 engages Trp680 in a manner similar to GSK. BT11 also established a salt bridge between its carboxylate group and Lys676, as well as two π–stacking interactions with Phe675 and Tyr705.

Compound T18, in which the benzothiophene is replaced by a single thiophene ring connected to the benzoic acid fragment, adopts a conformation similar to BT11 within the TRPC6 agonist cavity (Figure 5e). T18 forms three hydrogen bonds with Glu672, Trp680, and Asn702, in addition to a salt bridge with Lys676 and a π–stacking interaction with Tyr705 (Figure 5f). However, unlike BT11, T18 does not engage in π–stacking interactions with Phe675. Its XP GScore is −11.515 kcal/mol, which is comparable to and slightly higher than that of BT11 (−11.452 kcal/mol).

For compound INV29, in which the positions of the naphthalene and benzothiophene groups are swapped, the overall conformation is similar to the other derivatives. Within the TRPC6 agonist cavity, INV29 adopts an angle of less than 90° between the naphthalene group and the benzoic acid fragment (Figure 5g). INV29 forms four hydrogen bonds with Glu672, Trp680, Asn702, and Tyr705, in addition to a salt bridge with Lys676 and a π–stacking interaction with Phe675 (Figure 5h). Thus, its interaction profile is as complete as that of BT11, although its XP GS score is slightly lower (−11.226 kcal/mol).

To assess the importance of the phenolic hydroxyl group, we performed docking of compound SOH95, which lacks this functional group. SOH95 adopts a conformation similar to BT11 (Figure 5i). In the 2D interaction map (Figure 5j), SOH95 shows fewer interactions compared with its congeners. The compound retains a hydrogen bond with Trp680 (a key interaction), a salt bridge with Lys676, and a π–stacking interaction with Phe675. SOH95 showed the least favorable docking score in the series (−9.871 kcal/mol). Nevertheless, since its binding mode is similar to the other compounds and it preserves the interaction with Trp680, SOH95 remains an interesting candidate for further investigation by molecular dynamics simulations.

**Figure 6:**
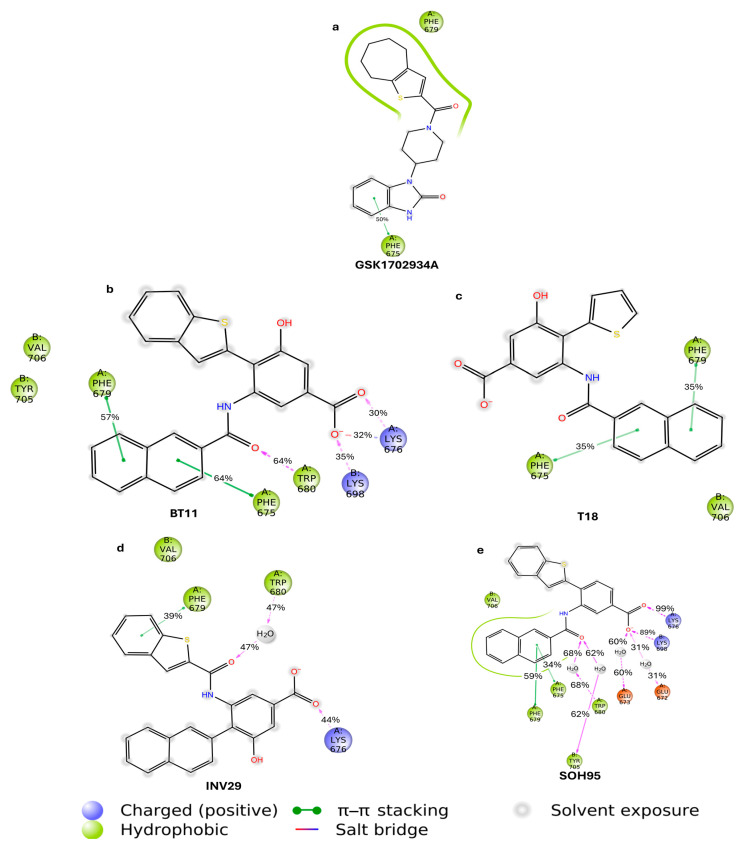
Residence times of the most relevant interactions for GSK (**a**), BT11 (**b**), T18 (**c**), INV29 (**d**), and SOH95 (**e**). Only residue interactions that persisted for more than 30% of the total simulation time (100 ns) are shown. Hydrophobic amino acids are shown in green, cationic residues in blue, and anionic residues in red.

To evaluate the stability of the designed molecules within the binding cavity and their interactions with key residues, we performed 100 ns molecular dynamics simulations for each compound. One of the most informative ways to visualize the results of such simulations is through graphical representations of ligand–protein intermolecular interaction lifetimes. These plots report the fraction of the simulation during which each interaction persisted (Figure 6a–e).

In Figure 6a, compound GSK is stabilized mainly through hydrophobic contacts in the vicinity of Phe679. The hydrogen bond with Trp680 does not persist for more than 30% of the simulation time. However, a π–stacking interaction with Phe675 is maintained for approximately 50% of the trajectory.

Figure 6b shows the interaction residence times for the hit compound BT11. This ligand displayed the most consistent interaction profile over time. The hydrogen bond with Trp680 persisted for 64% of the simulation. The naphthalene fragment engaged in two π–stacking interactions with Phe675 and Phe679, both lasting more than 57% of the time. The carboxylate group formed hydrogen bonds and a salt bridge with Lys676 and Lys698 for nearly 100% of the simulation. These interactions appear to serve as anchors within the agonist cavity. We propose that the benzoic acid fragment could represent an important pharmacophore for the design of new potential TRPC6 agonist series. The success of such designs would depend on their ability to also establish the critical hydrogen bond with Trp680.

In Figure 6c, corresponding to compound T18, the removal of the benzene ring results in greater solvent exposure. This leads to the loss of interactions with Lys676, Lys698, and the amide carbonyl–Trp680 hydrogen bond. Nonetheless, T18 preserves the same π–stacking interactions with Phe675 and Phe679 as BT11.

For compound INV29 (Figure 6d), the exchange of the benzothiophene and naphthalene rings proved unfavorable, consistent with the predominantly water-mediated H-bond to Trp680. The compound interacts with Trp680 for 47% of the simulation time, but only through a water-mediated hydrogen bond. We propose that the affinity of this derivative is highly dependent on the type of ring connected to the amide group.

Finally, in the case of SOH95 (Figure 6e), the absence of the hydroxyl group results in greater water penetration into the cavity. Although the hydroxyl group was not critical for direct intermolecular interactions with the channel (Figure 6b), it may play an important entropic role. Interactions with Lys676 and Lys698 were preserved, while the Trp680 hydrogen bond was maintained 68% of the time through a water-mediated interaction. Interestingly, two additional water-mediated interactions were formed with Gly672 and Gly673.

**Figure 7:**
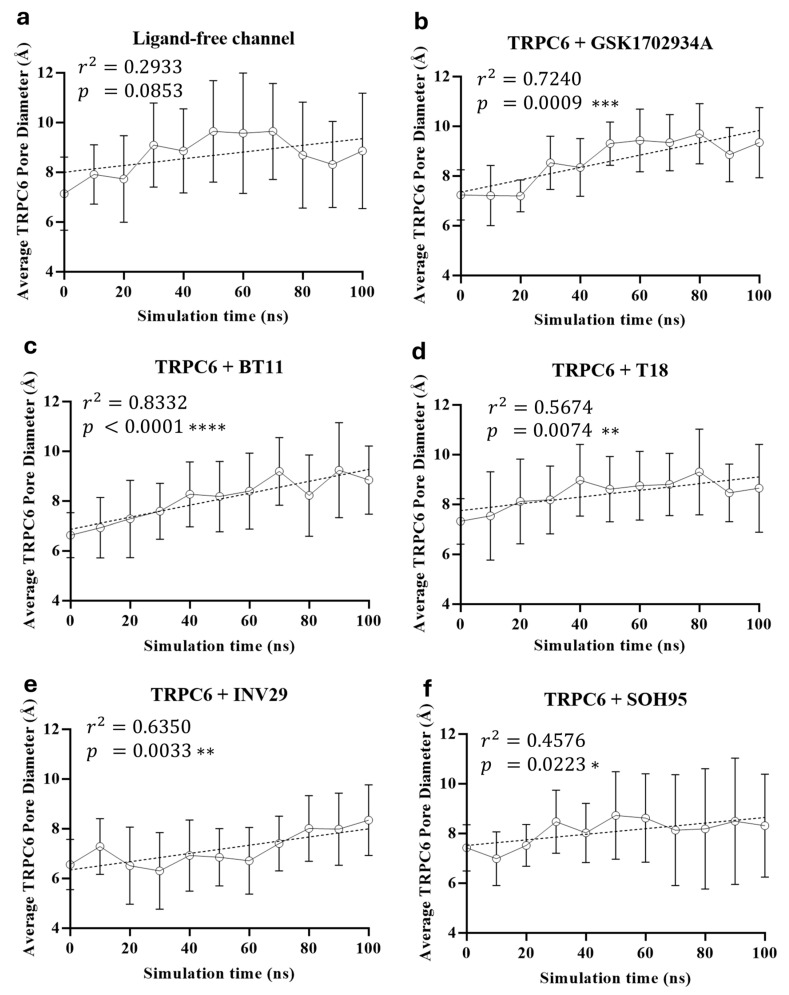
Time evolution of the average TRPC6 pore diameter (Å) during 100 ns molecular dynamics simulations. (**a**) Negative control: ligand-free TRPC6 channel. (**b**) Positive control: TRPC6 channel bound to GSK1702934A. (**c**) TRPC6 + BT11. (**d**) TRPC6 + T18. (**e**) TRPC6 + INV29. (**f**) TRPC6 + SOH95. Each plot shows the regression line, the coefficient of determination (r^2^), and the statistical significance (*p*-value). Vertical error bars represent the standard deviation of the average pore diameter values calculated at each 10 ns interval. Statistical significance: *p* < 0.05 (*), *p* < 0.01 (**), *p* < 0.001 (***), *p* < 0.0001 (****). The pore diameter was estimated as the distance between residues of the selectivity filter (Phe683 and Gly684, using the carbonyl groups of their backbones) and the gating residues (Leu723, Ile727, and Phe731, using their side chains).

To further evaluate the agonist potential of BT11 and its derivatives in TRPC6, we analyzed pore expansion dynamics with each ligand, as well as in the absence of ligands. Figure 7 shows the changes in pore diameter over the course of the simulations. In the ligand-free system (Figure 7a), no statistically significant pore expansion was observed, as indicated by the absence of any trend toward increased pore diameter. Between 30 and 80 ns, the pore remained stable, and after 80 ns, the diameter even decreased. In contrast, the reference agonist GSK (Figure 7b) promoted a linear pore expansion over time, with *p* = 0.0009 and r^2^ = 0.7240. BT11 (Figure 7c) also produced a linear pore expansion (r^2^ = 0.8332) and exhibited the strongest statistical significance (*p* < 0.0001). Compounds T18 (Figure 7d) and INV29 (Figure 7e) showed weaker agonist capacity than BT11, with less linear expansion trends and lower statistical significance. Compound SOH95 (Figure 7f) yielded the lowest statistical significance in the series, although its behavior was comparable to T18 and INV29. Based on these results, we propose that BT11 exhibits a TRPC6 agonist profile, with a pore expansion capacity comparable to or greater than that of GSK.

**Figure 8:**
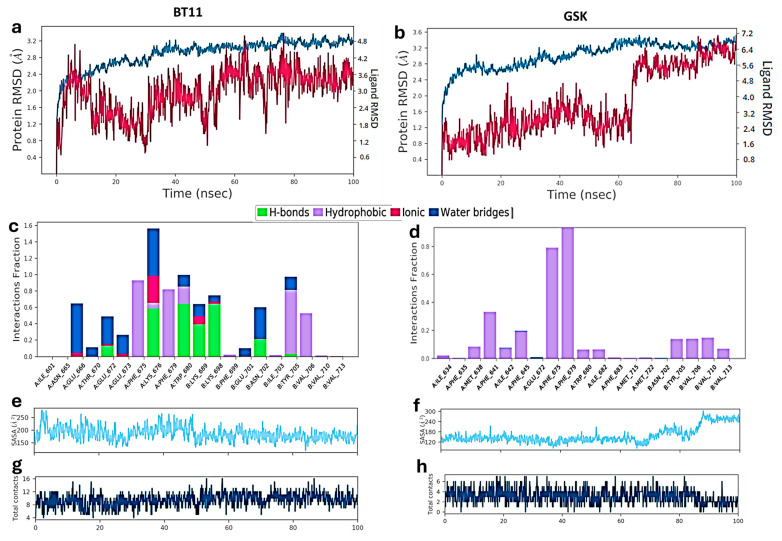
Results from molecular dynamics simulations (Isothermal–isobaric ensemble with constant Number of particles, Pressure, and Temperature (NPT ensemble), 1 atm, 310 K, 100 ns) for the hit compound BT11 (**left**) and the reference agonist GSK (**right**). (**a**,**b**) RMSD plots for the TRPC6 channel alone (blue) and for the channel–ligand complex (red). (**c**,**d**) Fraction of interactions per residue: green bars, hydrogen bonds; purple bars, hydrophobic contacts; magenta bars, ionic interactions; blue bars, water-mediated hydrogen bonds. (**e**,**f**) SASA plots. (**g**,**h**) Total ligand–protein contact plots. BT11 displays a higher fraction of stabilizing interactions compared to GSK, mediated mainly by hydrogen bonds and salt bridges. The solvent-exposure pattern is also more favorable for BT11, as reflected by a decrease in SASA after 60 ns, whereas GSK shows an increase. Finally, BT11 established a greater number of total contacts with the protein (*Y*-axis scale: 4–16, average ~10) than GSK (*Y*-axis scale: 0–6, average ~4).

Figure 8 compares the Root-mean-square deviation (RMSD), fraction of interaction per residue, Solvent-accessible surface area (SASA), and total contacts between BT11 and GSK. The BT11-TRPC6 complex (Figure 8a) showed strong RMSD value fluctuations, stabilizing around 50 ns at an average value of 3.6 Å. These fluctuations could be attributed to the inherent dynamics of the ionized carboxylate group and its interactions with Lys676 and Lys698 residues. On the other hand, the GSK compound displayed more stable RMSD until approximately 60 ns (Figure 8b), which can be partly attributed to its lower number of rotatable bonds. However, the compound experienced an abrupt change in RMSD value, rising to around 7 Å from 60 to 100 ns of simulation, possibly due to water entry into the agonist cavity.

A residue-wise interaction analysis (Figure 8c,d) further highlights the superior binding profile of BT11 compared to GSK. BT11 stabilizes the complex mainly through hydrogen bonds and ionic interactions (green and magenta bars, respectively), while GSK relies predominantly on hydrophobic contacts (purple bars). Importantly, the interaction of BT11 with Trp680 is sustained by a hydrogen bond for a substantial fraction of the trajectory (Figure 8c), whereas GSK engages Trp680 primarily through hydrophobic contacts that persist for less than 10% of the total simulation time (Figure 8d).

The SASA analysis also supports these findings. BT11 maintains a consistent SASA throughout the simulation (Figure 8e), whereas GSK exhibits a marked increase in SASA after 60 ns (Figure 8f), suggesting progressive water penetration into the binding cavity. Finally, BT11 clearly outperforms GSK in the total number of protein–ligand contacts (Figure 8g,h), with an average of 10 contacts compared to only 4 for GSK.

To obtain a more quantitative comparison of the binding affinity among the designed derivatives, we performed a Molecular Mechanics/Generalized Born Surface Area (MM-GBSA) energy calculation (Table 2).

**Figure 9:**
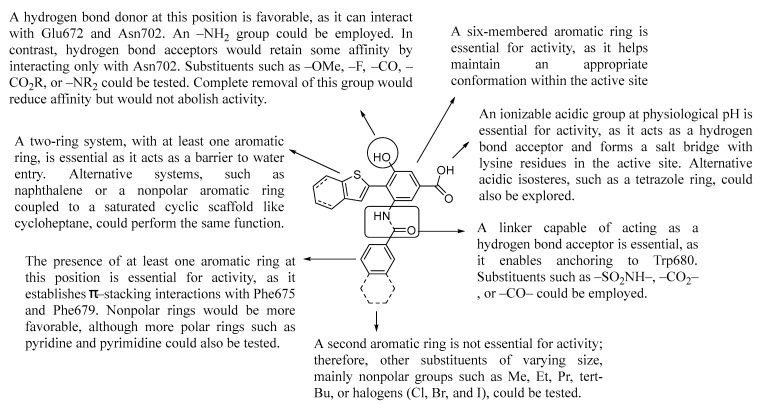
Summary of the proposed fragments and modifications for the obtention of new benzoic acid derivatives with potential affinity for TRPC6.

Based on the results obtained (Table 2), the hit compound BT11 showed a consistent binding energy of −67.72 kcal/mol, achieving the lowest value among all systems, indicating the highest affinity for the channel. In the case of GSK, although its binding energy increased by approximately 12 kcal/mol, it remained favorable, suggesting that GSK also retains affinity for the channel—lower than BT11 but higher than T18 and INV29, both of which exhibited an energy increase. For T18, the energy rose by 21.2 kcal/mol, reaching −50.65 kcal/mol, the weakest affinity of all compounds. INV29 showed an increase of 12.57 kcal/mol, comparable to GSK, indicating a similar affinity for GSK and higher than T18. Finally, SOH95 displayed a net increase of −3.84 kcal/mol from the initial frame, resulting in a final value of −56.83 kcal/mol, almost equal to the value of GSK. These results are consistent with the molecular dynamics simulations, where BT11 exhibited the best behavior, followed by SOH95, GSK, INV29, and T18—the same order observed in the binding energy calculations. Because TRPC3/6/7 share structural similarity around the S6–pore helix agonist region, BT11 might also modulate TRPC3/7; establishing isoform selectivity for BT11 and its analogs will require dedicated experimental profiling beyond the scope of the present in silico work. Figure 9 shows a summary of the main structural ideas for the design of new benzoic acid derivatives, proposed as TRPC6 ligands. A benzoic acid bearing a hydrogen bond acceptor function at the *meta*-position is suggested as the pharmacophore for the development of new TRPC6 ligands.

## 3. Materials and Methods

### 3.1. Molecular Docking

The cryo-EM structure of human TRPC6 was obtained from the Protein Data Bank, PDB ID = 6UZ8 [26]. This homotetrameric channel comprises residues 85–931 in each subunit (A–D) at 2.84 Å resolution, with the best available PDB validation profile. The structure was first curated by removing lipids and exogenous molecules. Protein preparation was performed with the Protein Preparation Wizard (Schrödinger Release 2023-2, Schrödinger, LLC, New York, NY, USA, 2021) [35]. Protonation/tautomeric states were assigned with Epik [36] at pH 7.4 ± 0.1, and missing side chains present in the sequence were rebuilt with Prime [37]. Hydrogen-bond networks and protonation of some residues (Ser, Thr, Tyr, Cys, Asn, Gln, His) were optimized using PROtein pKa prediction (PROPKA) at pH 7.4. The structure was then minimized to a heavy-atom RMSD convergence of 0.30 Å under the OPLS3e force field [38]. Remaining exogenous molecules (waters/solvents/contaminants) were removed after visual inspection.

Ligands were prepared with LigPrep. Low-energy conformers were generated under OPLS3e, and plausible ionization states were enumerated with Epik at pH 7.4 ± 0.1. All entries were manually checked to confirm correct optimization, stereochemistry, and valence.

Docking was performed with the Induced Fit Docking (IFD) module [39] using a standard protocol (up to 20 poses/ligand) and OPLS3e force field. The receptor grid was centered on the cocrystallized AM-0883 ligand at the A-pore/B-S6 interface. Limited ring conformational sampling was allowed for ring flips/torsions within 2.5 kcal/mol of the lowest-energy conformer. Nonplanar amide torsions were penalized, and planarity was enforced for π-conjugated groups (e.g., aromatic rings). Glide [40] was used with van der Waals scaling factors of 0.50 for both the receptor and ligand to enhance flexibility and enable optimal pose sampling (maximum 20 poses/ligand). Residues within 5 Å of each pose were locally refined. Poses within 30 kcal/mol of the best pose and ranking within the top 20 were redocked. Scoring employed extra-precision (XP) mode [41] and associated descriptors; more negative scores indicate more favorable binding.

### 3.2. Initial Virtual Screening

An initial screen included 103 in-house molecules spanning five major scaffolds with shared structural features, selected by the following criteria: (i) 3–5 aromatic rings; (ii) polar linker (ester, amide, sulfonamide, ether, or amine); (iii) molecular weight < 500 Da. The docking protocol for each derivative was the same as that described in the previous section for IFD. In the Supplementary Materials, we present the structural cores of five scaffold families that were docked, as well as a table showing the XP GScore values for the best compound of each evaluated series. Based on these results, we initiated iterative design–docking cycles to introduce new interactions in the active site guided by cavity geometry and residue proximity, adding 95 further analogs. In addition, the four known TRPC6 agonists were docked for benchmarking, yielding a total of 202 compounds evaluated.

### 3.3. Molecular Docking Validation

We evaluated the discriminative performance of the docking protocol using a benchmarking set of N = 500 unique compounds (10 TRPC6 actives with experimental evidence and 500 decoys). Decoys were derived from the Chemical Database of Bioactive Molecules (ChEMBL) v36 database (initial pool = 440,050 candidates) and selected to be property-matched yet topologically dissimilar to the actives. Briefly, for each active, we searched the candidate pool for molecules that satisfied two layers of criteria:

- Molecular Weight (MW), calculated Logarithm of the partition coefficient (cLogP), Topological Polar Surface Area (TPSA): within ±10% of the active’s value (continuous properties).
- Hydrogen Bond Donors (HBD) and Hydrogen Bond Acceptors (HBA): within ±1 of the active’s integer counts.
- Topological dissimilarity. Molecular fingerprints were computed as Morgan Extended-Connectivity Fingerprints (ECFP-like) with radius = 2 and 2048 bits; candidates were required to have Tanimoto similarity ≤ 0.40 to the active (structural dissimilarity constraint).

Fifty decoys per active were selected, prioritizing those with the smallest property-distance to ensure close property matching while preserving low structural similarity. This procedure yielded 500 decoys for the 10 actives used in the enrichment benchmark.

The best pose per compound (most favorable docking score) was retained to build a per-compound ranking. Because docking scores are reported as negative energies, values were sign-inverted so that larger values indicate a more favorable ranking. Performance was quantified using standard virtual screening metrics: ROC-AUC (global discrimination), PR-AUC (robust under class imbalance), and early-recognition enrichment factors EF1%, EF2%, and EF5% (density of actives within the top 1–5% of the ranked list). 95% confidence intervals for ROC-AUC and EF metrics were estimated by bootstrap resampling (2000 replicates).

### 3.4. Molecular Dynamics Studies

#### 3.4.1. Starting Structures

For each system, the initial state (t = 0) was the best-scoring docking pose (most negative score) of the ligand. We simulated the four top-ranked derivatives, the highest-scoring reference agonist (GSK), and the apo TRPC6 channel.

#### 3.4.2. System Setup

Systems were prepared with the System Builder in Desmond. We used a minimized cubic periodic box large enough to fully contain the protein. Solvation employed the Transferable Intermolecular Potential 3-Point water model (TIP3P). The protein was embedded in a 1-palmitoyl-2-oleoyl-sn-glycero-3-phosphoethanolamine (POPE) lipid bilayer at 310 K, with the membrane automatically positioned to cover the transmembrane domain, as described in the literature [26]. Counterions (Na^+^) were added to neutralize the net charge, and 0.15 M NaCl was included to mimic physiological ionic strength. All systems used the Optimized Potentials for Liquid Simulations, version 3e (OPLS3e) force field. For membrane positioning independent of specific simulation software, the transmembrane residue report for the TRPC6 channel from the Orientations of Proteins in Membranes (OPM) database can be consulted [42]. In the Supplementary Materials, we provide comparative information on membrane positioning performed by OPM versus the System Builder in Desmond. The positions are quite similar; however, the trajectory stabilization achieved using the default membrane positioning option yielded more stable trajectories than the OPM-based positioning.

#### 3.4.3. Simulation Protocol

Molecular dynamics simulations were performed with the Molecular Dynamics module in Desmond. Each trajectory was run for 100 ns under NPT conditions at 310 K and 1 atm. Trajectories were recorded every 100 ps, yielding ~1000 frames per system.

#### 3.4.4. Interaction and Trajectory Analyses

Protein and ligand RMSD were computed with respect to their starting conformations. Ligand–residue interactions within the binding site were quantified using Desmond’s Simulation Interaction Diagram (SID), and their residence times were reported as the percentage of the total simulation. We also assessed total ligand–protein contacts, SASA, and inspected the system trajectory for qualitative consistency.

#### 3.4.5. Pore-Diameter Analysis

To monitor pore expansion over time, we measured distances between five residues critical for selectivity and gating: Phe683 and Gly684 (backbone carbonyls; cation selectivity filter) and Leu723, Ile727, Phe731 (side chains; pore seal). The same atoms were used as reference points in each subunit. Diameters were measured across A–C and B–D subunit pairs at t = 0 and every 10 ns, and the average pore diameter (Å) was plotted versus simulation time (ns). The apo (ligand-free) channel served as the negative control; GSK-bound TRPC6 served as the positive control.

#### 3.4.6. Molecular Dynamics Validation

For each protein–ligand complex (TRPC6 with the four candidates and with the reference ligand), we ran two independent 100 ns replicas starting from the same minimized pose but with distinct random seeds for velocity assignment and stochastic thermostats/barostats. Systems were built in a POPE bilayer, neutralized at 0.15 M, and equilibrated prior to production. Convergence was assessed by analysis of RMSD, SASA, and total ligand–protein contacts. Key interaction distances (e.g., Trp680 H-bond, Lys676/Lys698 salt bridge, Phe675/Phe679 π-stacking) were reported as contact residence (%) across replicas. In the Supplementary Materials, we present a comparison of the standard metric plots for the simulations in each replicate.

### 3.5. Statistics

Descriptive statistics were applied. Variable distributions were assessed with the Kolmogorov–Smirnov (KS) test. Parametric variables are reported as mean ± standard deviation. Pearson’s correlation was used for correlation analyses. Statistical significance was set at *p* < 0.05. All statistical analyses were performed in GraphPad Prism 9.5.1 (GraphPad Software, San Diego, CA, USA).

## 4. Conclusions

Leveraging an integrated structure-based workflow—docking, 100 ns molecular dynamics, and MM-GBSA—we rationally ranked benzoic-acid–derived TRPC6 agonists and identified BT11 as the lead candidate. BT11 is characterized by a hydrogen bond to Trp680 (64%), salt bridges to Lys676/Lys698 (~100% combined residence), π–stacking with Phe675/Phe679, a more stable complex RMSD, a higher contact count (~10 vs. 4 for GSK), and a decreasing SASA over time. Energetically, BT11 (−67.72 kcal/mol) was the strongest binder; SOH95 (−56.83 kcal/mol) was almost equal to GSK (−57.90 kcal/mol), while T18 (−50.65 kcal/mol) was the weakest. The SAR indicates that a meta-hydrogen-bond acceptor together with π–stacking capacity and salt-bridge formation are key drivers of affinity. Collectively, these findings nominate BT11 for experimental validation (e.g., Trp680/Lys676 mutagenesis and electrophysiology), guide the synthesis of next-generation analog series, and suggest that BT11 (and related analogs) could serve as potential therapeutic leads for autism spectrum disorder via TRPC6 activation.

## Figures and Tables

**Table 1:** Virtual screening performance of the docking protocol on TRPC6 actives versus property-matched decoys ^a^.

Metric	Value	CI95_lo	CI95_hi	TopK	Actives in TopK
ROC-AUC	0.984	0.964	0.998		
EF1%	37.36	18.68	70.05	5	4
EF2%	28.02	15.57	42.56	10	6
EF5%	17.51	9.73	19.46	24	9
PR-AUC	0.703				

^a^ ROC-AUC: area under the receiver operating characteristic curve; the probability that a randomly chosen active is ranked above a randomly chosen decoy. PR-AUC: area under the precision–recall curve; summarizes precision as a function of recall and is appropriate under class imbalance. EF1%/EF2%/EF5%: early-recognition enrichment factors; ratio of the observed fraction of actives in the top 1%/2%/5% of the ranked list to the fraction expected at random. TopK: number of compounds included in the top x% cutoff used to compute EFx%. Actives in TopK: count of true actives retrieved within TopK. CI95: bootstrap 95% confidence interval around the reported metric.

**Table 2:** MM-GBSA binding energies (ΔG, kcal/mol) for the designed derivatives.

GSK			BT11			T18			INV29			SOH95		
Frame No.	Time (ns)	ΔG	Frame No.	Time (ns)	ΔG	Frame No.	Time (ns)	ΔG	Frame No.	Time (ns)	ΔG	Frame No.	Time (ns)	ΔG
Docking Pose		−70.37			−66.73			−71.85			−67.68			−60.67
761	76	−52.91	861	86	−61.83	611	61	−47.99	861	86	−52.55	811	81	−55.00
771	77	−54.78	871	87	−76.04	621	62	−50.23	871	87	−48.42	821	82	−54.87
781	78	−58.23	881	88	−67.97	631	63	−50.98	881	88	−52.7	831	83	−53.24
791	79	−52.21	891	89	−63.99	641	64	−47.87	891	89	−54.27	841	84	−62.14
801	80	−55.18	901	90	−73.13	651	65	−55.82	901	90	−56.09	851	85	−56.11
811	81	−64.12	911	91	−63.89	661	66	−48.86	911	91	−57.18	861	86	−51.45
821	82	−56.49	921	92	−66.96	671	67	−50.4	921	92	−59.73	871	87	−55.37
831	83	−71.15	931	93	−68.41	681	68	−53.06	931	93	−55.57	881	88	−60.48
841	84	−59.46	941	94	−69.6	691	69	−52.78	941	94	−58.96	891	89	−62.24
851	85	−54.42	951	95	−65.42	701	70	−48.48	951	95	−55.67	901	90	−57.39
Avg.		−57.90			−67.72			−50.65			−55.11			−56.83
		±5.82			±4.37			±2.58			±3.33			±3.69

## Data Availability

The original contributions presented in the study are included in the article and Supplementary Materials; further inquiries can be directed to the corresponding authors.

## Supplementary Materials

The following supporting information can be downloaded at: https://www.mdpi.com/article/10.3390/ph18101577/s1, Figure S1. Structural cores of the five generations of molecules tested in the initial screening. Gen = Generation. Table S1. XP GScore values from the initial docking screening. Only the values of the best pose for each compound with a score lower than –9.0 kcal/mol are shown. Table S2. XP GScore values from the reference agonists. Figure S2. Superposition of the AM-0883 compound present in the crystal structure (PDB ID: 6UZ8) shown in green, and the best redocked pose of the same compound obtained using the protocol from the present study (shown in gray). The RMSD value obtained was 0.498 Å, which represents a good result validating the methodology. Values below 2 Å are considered acceptable. Figure S3. Comparison of molecular dynamics metrics under the initial conditions and after activation of the random-seed function in Desmond. Figure S4. Comparison of the initial membrane positioning performed using the coordinates provided by OPM (https://opm.phar.umich.edu/proteins/5023) (left) versus the positioning generated by the Desmond System Builder (right) (PDB ID = 6UZ8). As shown, there are no major differences. In the present study, the system setup generated by the Desmond System Builder was used. Figure S5. Comparison of the membrane–TRPC6 system after the initial relaxation. It can be observed that the system assembled by Maestro’s System Builder (right) achieves a more uniform distribution of phospholipids compared to the initial system based on OPM coordinates (left). Figure S6. RMSD plot for the BT11–TRPC6 complex based on the system built using the membrane coordinates from OPM. The blue line represents the RMSD of the protein, and the red line represents that of the complex. The protein RMSD never reached equilibrium throughout the simulation time. Based on this result, we propose that the default model (Desmond’s System Builder) is superior to the one provided by OPM for 100-ns simulations.

## Abbreviations

The following abbreviations are used in this manuscript:

5-HT	5-hydroxytryptamine
Akt	Protein Kinase B
apo	Ligand-free
ASD	Autism Spectrum Disorder
AUC	Area Under Curve
BDNF	Brain-derived Neurotrophic Factor
CaMKIV	Calcium/Calmodulin-Dependent Protein Kinase IV
CI	Confidence Interval
cLogP	Calculated Logarithm of the Partition Coefficient
CREB	cAMP Response Element–Binding Protein
cryo-EM	Cryo-electron Microscopy
DAG	Diacylglycerol
DSM-5	*Diagnostic and Statistical Manual of Mental Disorders, 5th edition*
ECFP	Extended-Connectivity Fingerprints
EF, EF1%, EF2%, EF5%	Enrichment Factor at the Top 1%, 2%, or 5% of a Ranked List
GABA	Gamma-aminobutyric Acid
GSK	GSK1702934A
HBA	Hydrogen Bond Acceptors
HBD	Hydrogen Bond Donors
IFD	Induced Fit Docking
IGF-1	Insulin-like Growth Factor 1
iPSC	Induced Pluripotent Stem Cells
KS	Kolmogorov–Smirnov
MD	Molecular Dynamics
mGluR5	Metabotropic Glutamate Receptor 5
MM-GBSA	Molecular Mechanics/Generalized Born Surface Area
MW	Molecular Weight
NPT	Isothermal–Isobaric Ensemble with Constant Number of Particles, Pressure and Temperature
OPLS3e	Optimized Potentials for Liquid Simulations, Version 3e
OPM	Orientations of Proteins in Membranes
PDB	Protein Data Bank
POPE	1-palmitoyl-2-oleoyl-sn-glycero-3-phosphoethanolamine
PR-AUC	Area under the Precision–Recall Curve
PROPKA	PROtein pKa Prediction
PTPRD	Protein Tyrosine Phosphatase Receptor Delta
RMSD	Root-Mean-Square Deviation
ROC	Receiver Operating Characteristic
ROC-AUC	Area under the Receiver Operating Characteristic Curve
SAR	Structure–Activity Relationship
SASA	Solvent-Accessible Surface Area
SID	Simulation Interaction Diagram
TACA	The Autism Community in Action
TIP3P	Transferable Intermolecular Potential 3-Point water model
TPSA	Topological Polar Surface Area
TRPC6	Transient Receptor Potential Canonical Type 6
XP	Extra-Precision
XP GScore	Glide Extra-Precision Docking Score (Reported in kcal/mol)
