# Supplementary material for: Structure-Guided Discovery of Benzoic-Acid-Based TRPC6 Ligands: An Integrated Docking, MD, and MM-GBSA SAR Study: Potential Therapeutic Molecules for Autism Spectrum Disorder
Source: Pharmaceuticals (Basel). 2025 Oct 18;18(10):1577. doi: 10.3390/ph18101577 (PMC12566912; doi:10.3390/ph18101577)
Supplement: Supplementary file 1 [file pharmaceuticals-18-01577-s001.zip › pharmaceuticals-3883882-supplementary.pdf]

Supplementary material

Structure-Guided Discovery of Benzoic-Acid-Based TRPC6 Ligands: An Integrated Docking, MD, and MM-GBSA SAR Study: Potential Therapeutic Molecules for Autism Spectrum Disorder

Nicolás Ignacio Silva [1], Gianfranco Sabadini [2], David Cabezas [3], Cristofer González [2], Paulina González [2], Jiao Luo [2], Cristian O. Salas [1], Marco Mellado [4], Marcos Lorca [5], Javier Romero-Parra [6,*] and Jaime Mella [2,7,*]

1 Departamento de Química Orgánica, Facultad de Química y de Farmacia, Pontificia Universidad Católica de Chile, Santiago de Chile 7820436, Chile; nsilvas@estudiante.uc.cl (N.I.S.); cosalas@uc.cl (C.O.S.)
2 Instituto de Química, Facultad de Ciencias, Universidad de Valparaíso, Av. Gran Bretaña 1111, Valparaíso 2360102, Chile; gianfranco.sabadini@postgrado.uv.cl (G.S.); cristofer.gonzalez@postgrado.uv.cl (C.G.); paulina.gonzalezd@postgrado.uv.cl (P.G.); jiao.luo@postgrado.uv.cl (J.L.)
3 Departamento de Ciencias Biológicas y Químicas, Facultad de Ciencias, Universidad San Sebastián, Campus Los Leones, Lota 2465, Providencia, Santiago 7510157, Chile; dcabezasg@docente.uss.cl
4 Centro de Investigación en Ingeniería de Materiales, Universidad Central de Chile, Santiago 8330507, Chile; marco.mellado@ucentral.cl
5 Facultad de Ciencias de la Vida, Carrera de Química y Farmacia, Universidad Viña del Mar, Viña del Mar 2520000, Chile; marcos.lorca@uvm.cl
6 Organic Chemistry and Physical Chemistry Department, Faculty of Chemical and Pharmaceutical Sciences, Universidad de Chile, Olivos 1007, Santiago 7820436, Chile
7 Centro de Investigación, Desarrollo e Innovación de Productos Bioactivos (CInBIO), Universidad de Valparaíso, Av. Gran Bretaña 1111, Valparaíso 2360102, Chile
* Correspondence: javier.romero@ciq.uchile.cl (J.R.-P.); jaime.mella@uv.cl (J.M.)

**Figure S1.** Structural cores of the five generations of molecules tested in the initial screening. Gen = Generation.	2
**Table S1.** XP GScore values from the initial docking screening. Only the values of the best pose for each compound with a score lower than –9.0 kcal/mol are shown.	2
**Table S2.** XP GScore values from the reference agonists.	3
**Figure S2.** Superposition of the AM-0883 compound present in the crystal structure (PDB ID: 6UZ8) shown in green, and the best redocked pose of the same compound obtained using the protocol from the present study (shown in gray). The RMSD value obtained was 0.498 Å, which represents a good result validating the methodology. Values below 2 Å are considered acceptable.	4
**Figure S3.** Comparison of molecular dynamics metrics under the initial conditions and after activation of the random-seed function in Desmond.	5–9
**Figure S4.** Comparison of the initial membrane positioning performed using the coordinates provided by OPM (https://opm.phar.umich.edu/proteins/5023) (left) versus the positioning generated by the Desmond System Builder (right) (PDB ID = 6UZ8). As shown, there are no major differences. In the present study, the system setup generated by the Desmond System Builder was used.	10
**Figure S5.** Comparison of the membrane–TRPC6 system after the initial relaxation. It can be observed that the system assembled by Maestro's System Builder (right) achieves a more uniform distribution of phospholipids compared to the initial system based on OPM coordinates (left).	10
**Figure S6.** RMSD plot for the BT11–TRPC6 complex based on the system built using the membrane coordinates from OPM. The blue line represents the RMSD of the protein, and the red line represents that of the complex. The protein RMSD never reached equilibrium throughout the simulation time. Based on this result, we propose that the default model (Desmond's System Builder) is superior to the one provided by OPM for 100-ns simulations.	11

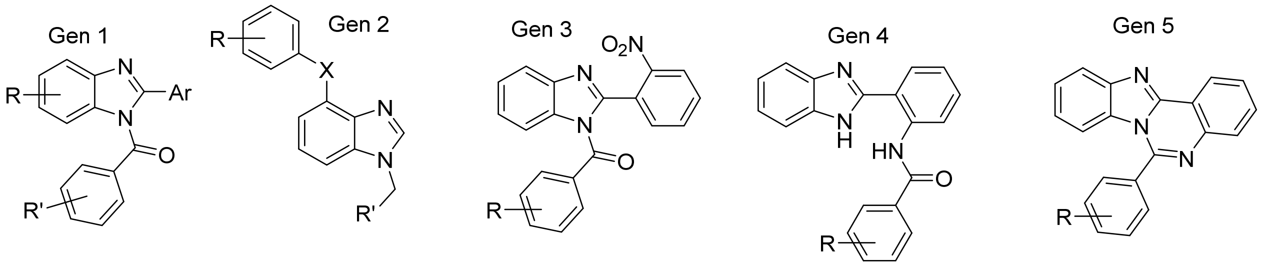

Figure S1. Structural cores of the five generations of molecules tested in the initial screening. Gen = Generation.

Table S1. XP GScore values from the initial docking screening. Only the values of the best pose for each compound with a score lower than –9.0 kcal/mol are shown.

Generation	Number of Compounds	R	Best XP GScore (kcal/mol)	Number of poses
1°	38	R = 5,6-Di-Me<br>Ar = Pyridine<br>R' = 2-Naphthalene	–9.601	15
	7	R = 5-F<br>Ar = Pyridine<br>R' = 1- Naphthalene	–9.089	8
2°	14	R = p-OH<br>X = -NH-<br>R' = cyclopentyl	–9.779	17
	13	R = p-OMe<br>X = -NH-<br>R' = cyclopentyl	–9.758	12
	12	R = p-OH<br>X = -NH-<br>R' = cyclopropyl	–9.380	16
	4	R = m,p-Di-Cl<br>X = -NH-<br>R' = cyclopropyl	–9.369	15
	3	R = p-Cl<br>X = -NH-<br>R' = cyclopropyl	–9.251	13
	16	R = p-OMe<br>X = -SO_2NH-<br>R' = cyclopropyl	–9.142	15
	15	R = p-OMe<br>X = - SO_2NH-<br>R' = -Phenyl	–9.142	15
3°		None		
4°	11	R = 4-Phenyl	–9.760	18
	12	R = 1- Naphthalene	–9.634	16
	10	R = 2,3,4-Tri-OMe	–9.422	17
	13	R = 2- Naphthalene	–9.246	18
5°	13	R = 2- Naphthalene	–11.065	12
	8	R = 4-$N(CH_3)_2$	–10.287	19
	7	R = 4-F	–9.720	16
	10	R = 2,3,4-Tri-OMe	–9.714	15
	3	R = 2,6-Di-F	–9.147	17

Table S2. XP GScore values from the reference agonists.

Type of molecule	Name	Structure	Best XP GScore (kcal/mol)	Number of poses
**Agonists**	(R)-AM-0883	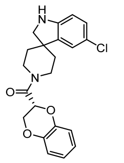	–9.513	19
	GSK1702934A	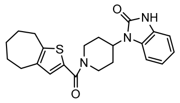	–9.202	13
	M085	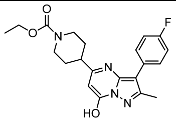	–7.850	19
	Hyperforin	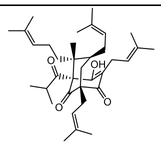	–6.223	16

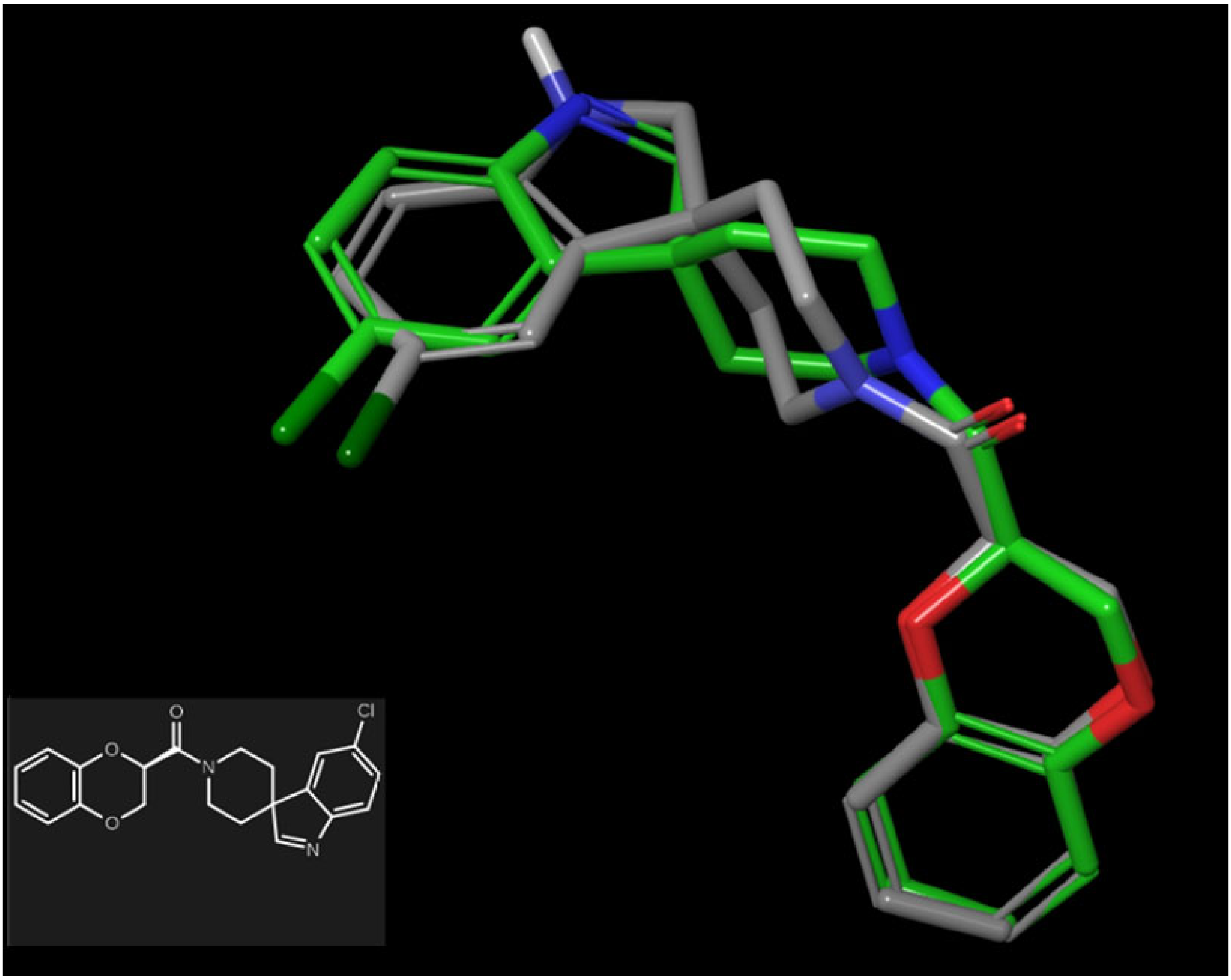

Figure S2. Superposition of the AM-0883 compound present in the crystal structure (PDB ID: 6UZ8) shown in green, and the best redocked pose of the same compound obtained using the protocol from the present study (shown in gray). The RMSD value obtained was 0.498 Å, which represents a good result validating the methodology. Values below 2 Å are considered acceptable.

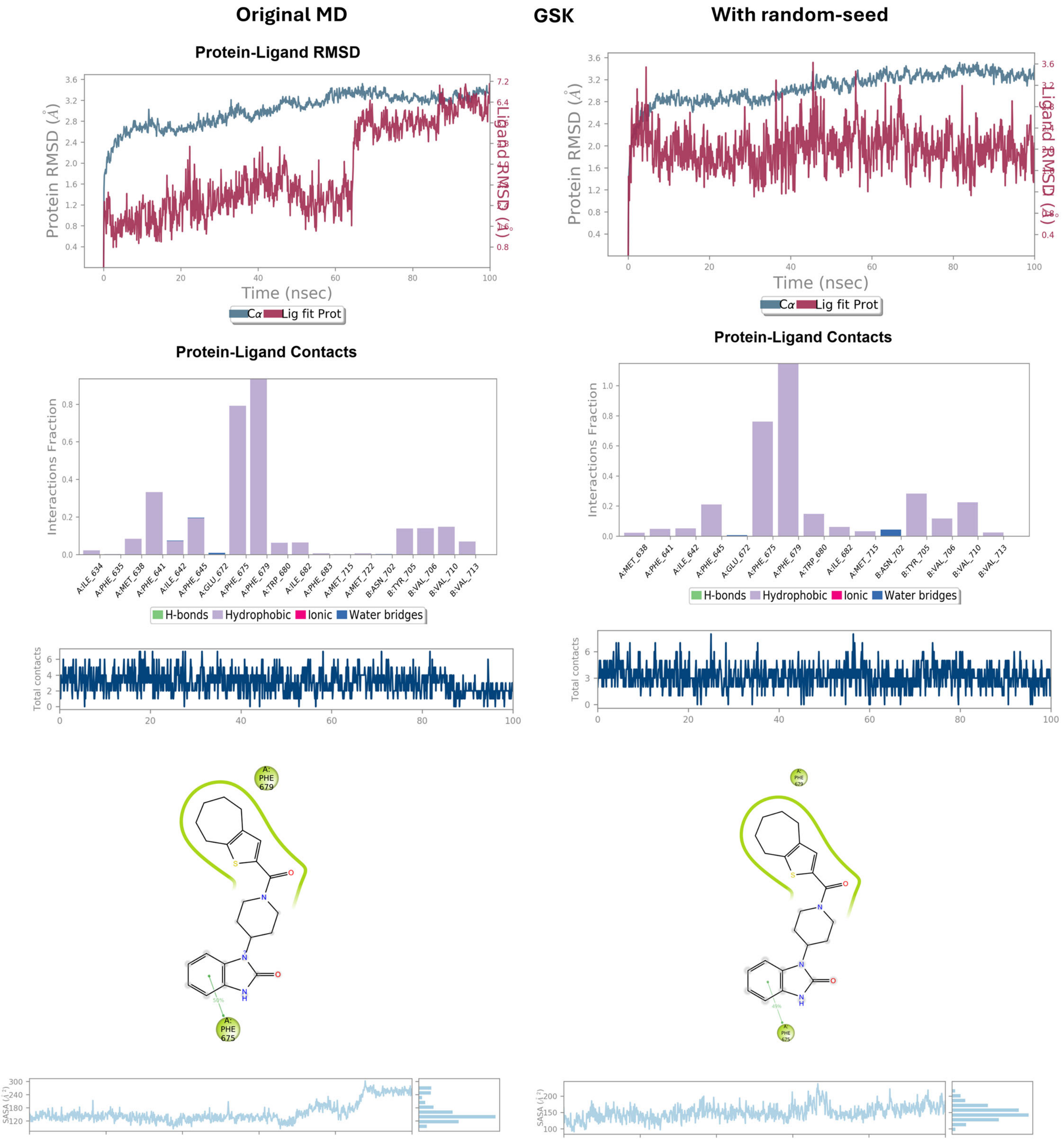

Figure S3. Comparison of molecular dynamics metrics under the initial conditions and after activation of the random-seed function in Desmond.

Original MD **BT11** **With random-seed**

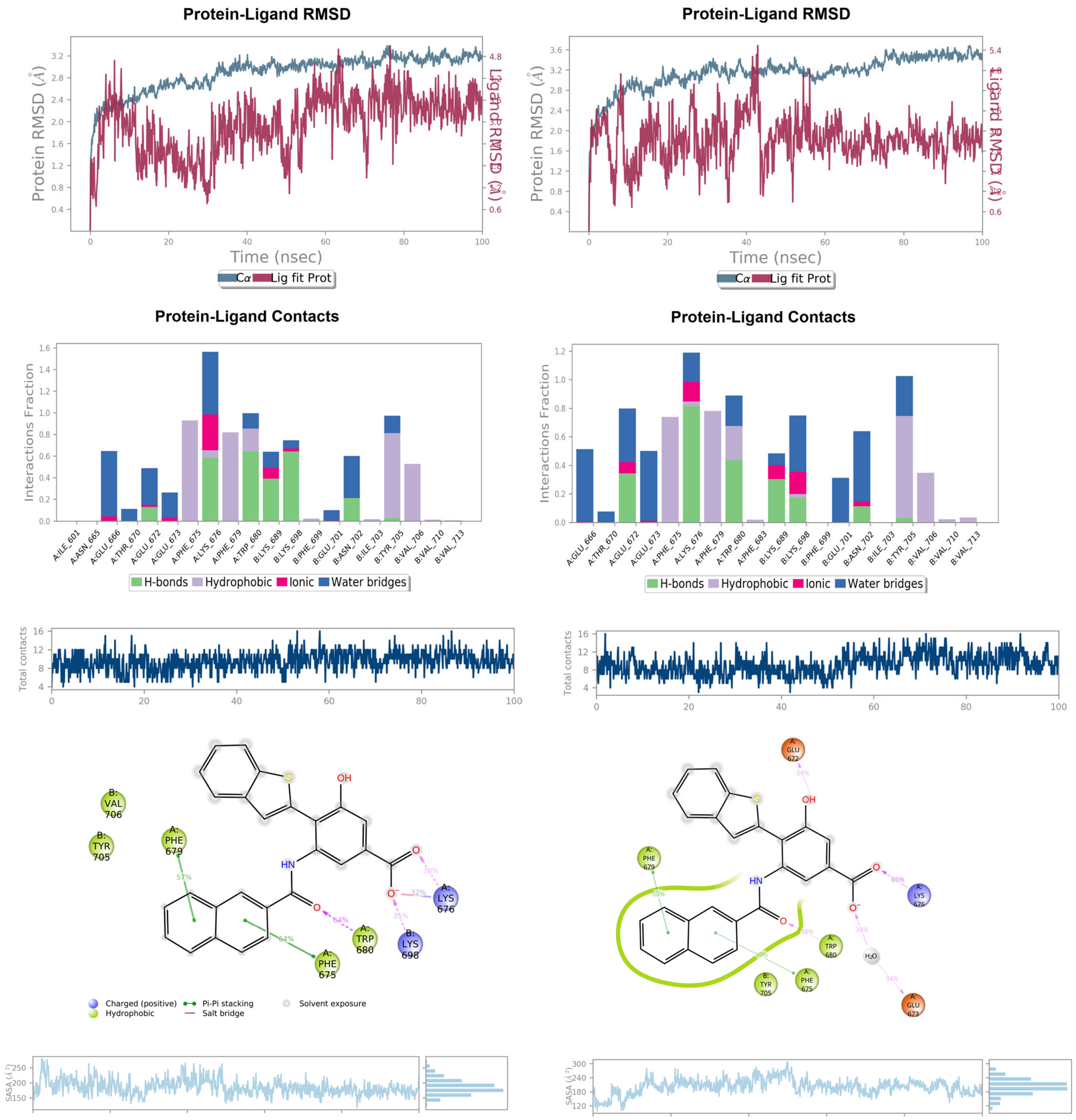

Figure S3. Cont.

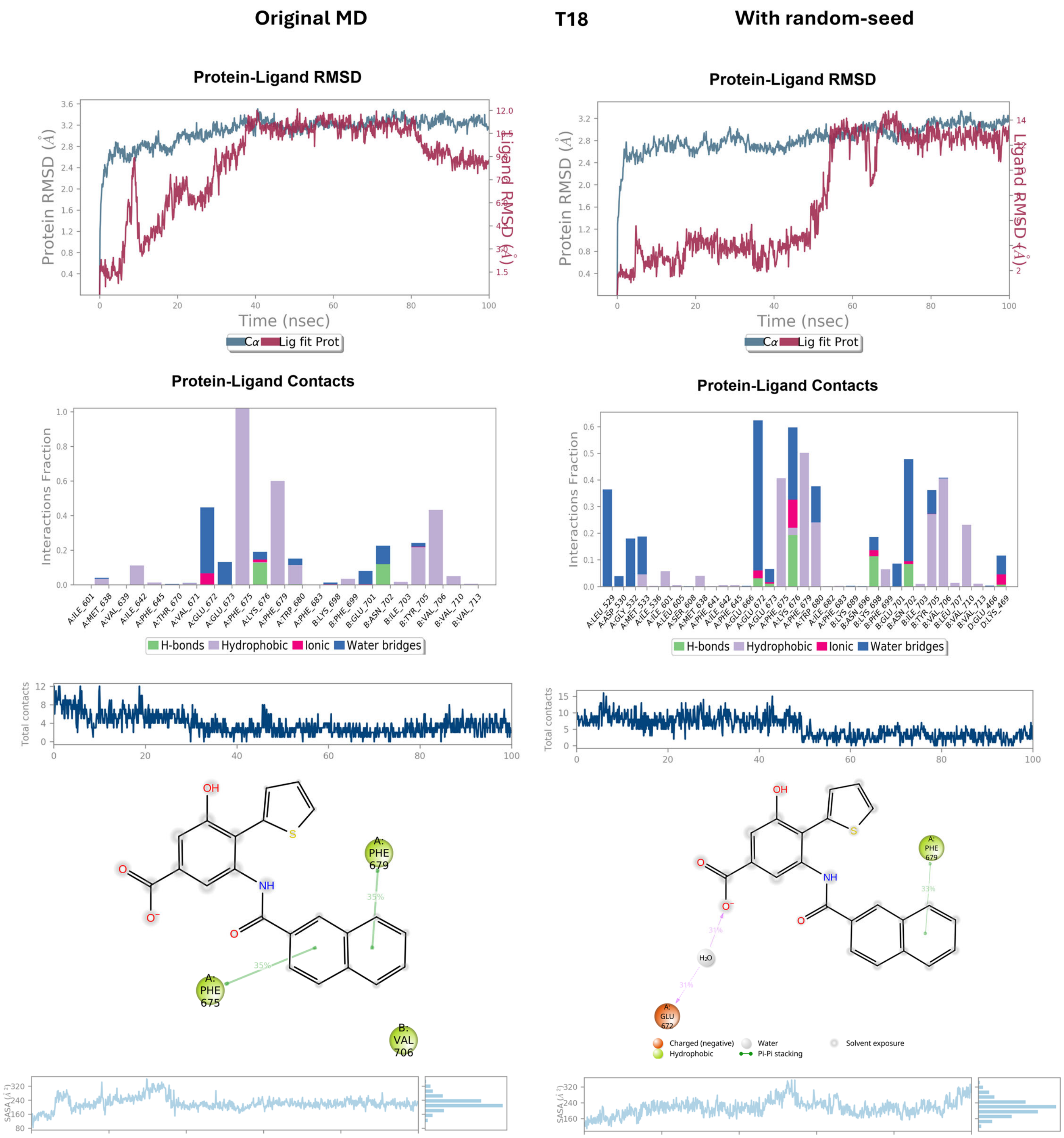

Figure S3. Cont.

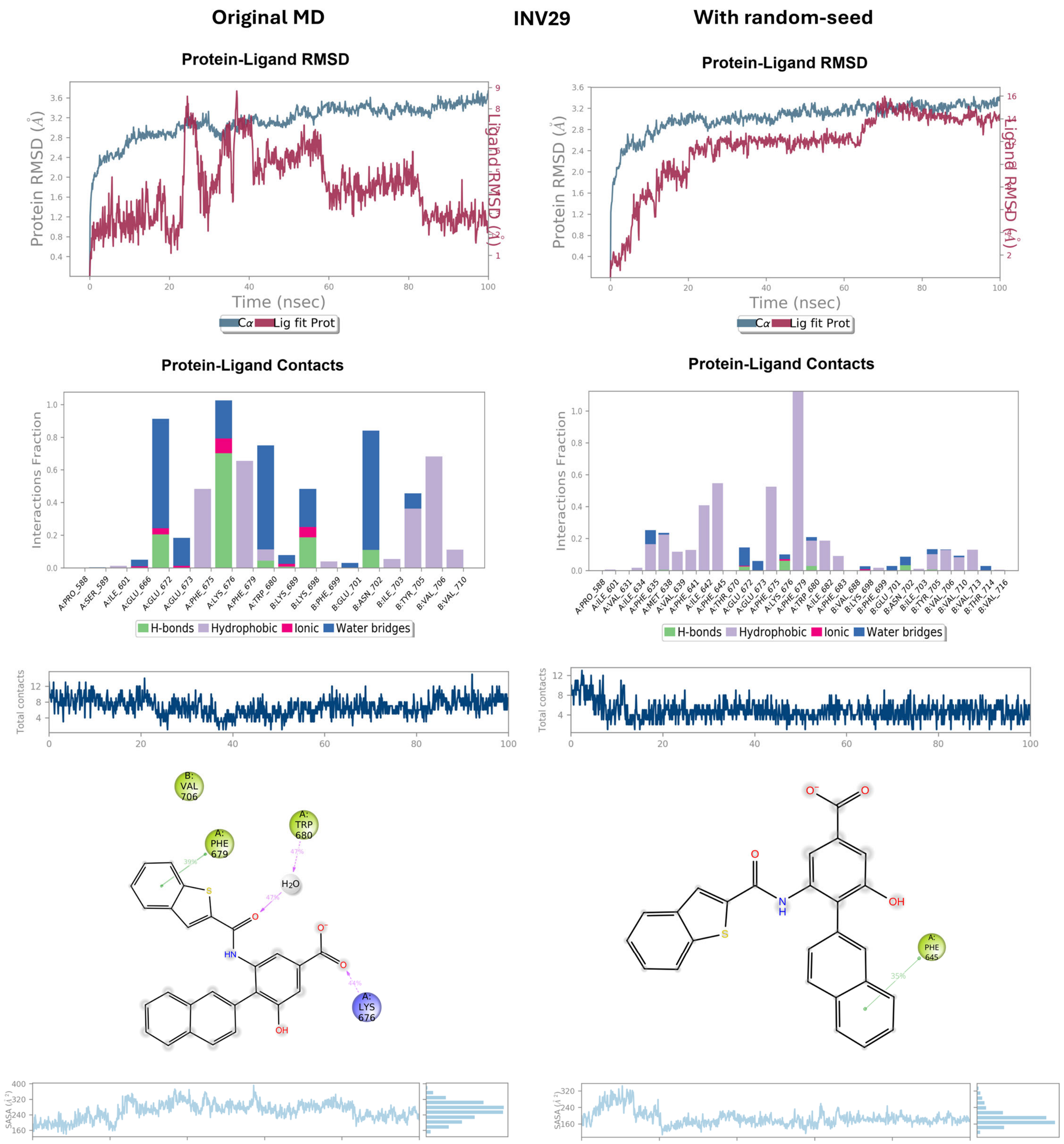

Figure S3. Cont.

Original MD **SOH95** **With random-seed**

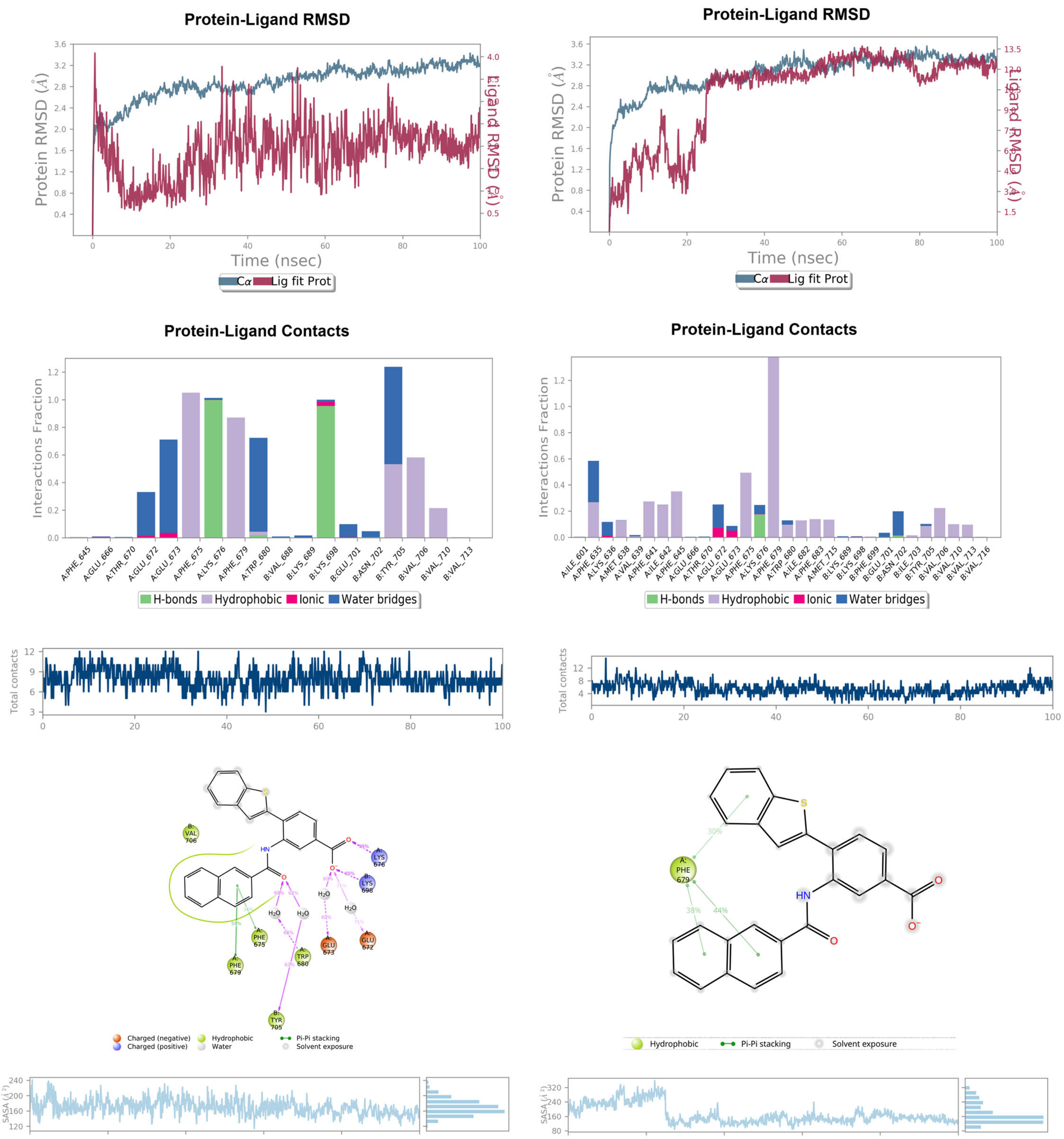

Figure S3. Cont.

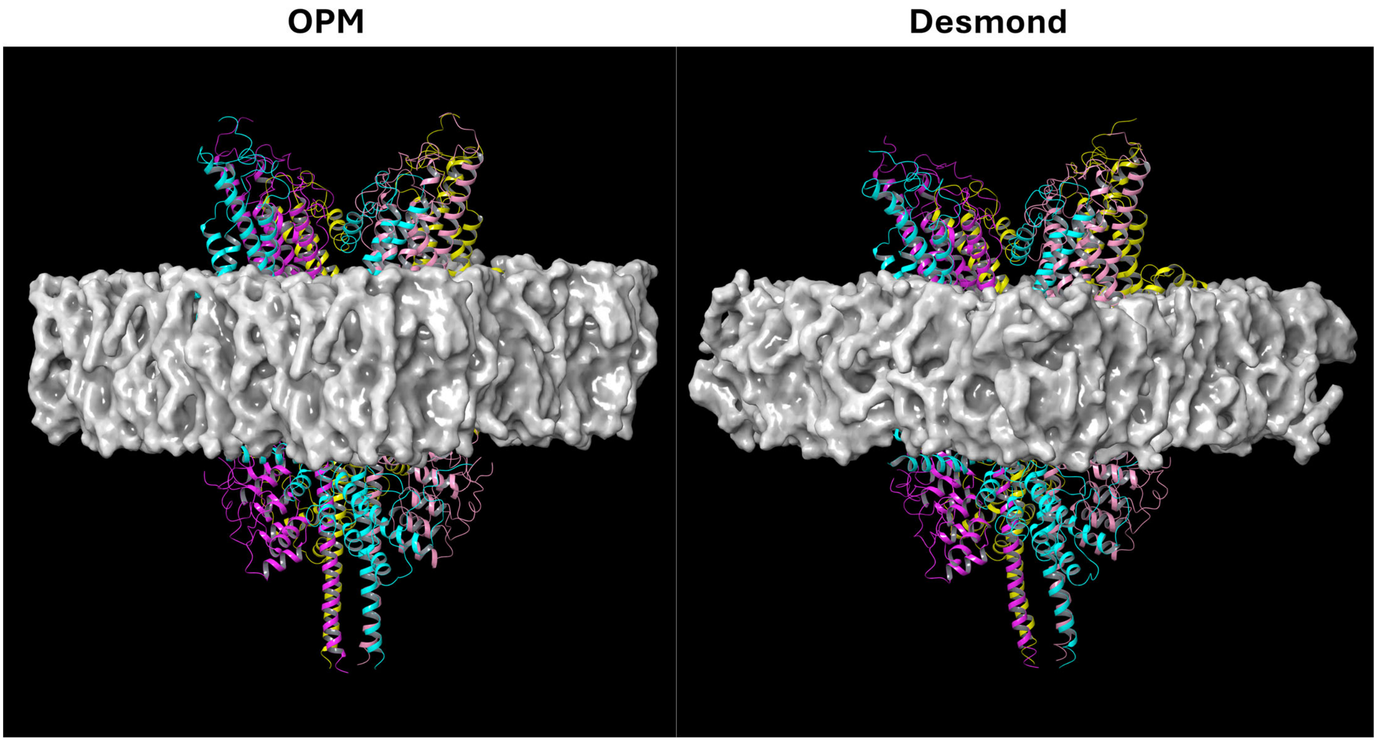

Figure S4. Comparison of the initial membrane positioning performed using the coordinates provided by OPM (https://opm.phar.umich.edu/proteins/5023) (left) versus the positioning generated by the Desmond System Builder (right) (PDB ID = 6UZ8). As shown, there are no major differences. In the present study, the system setup generated by the Desmond System Builder was used.

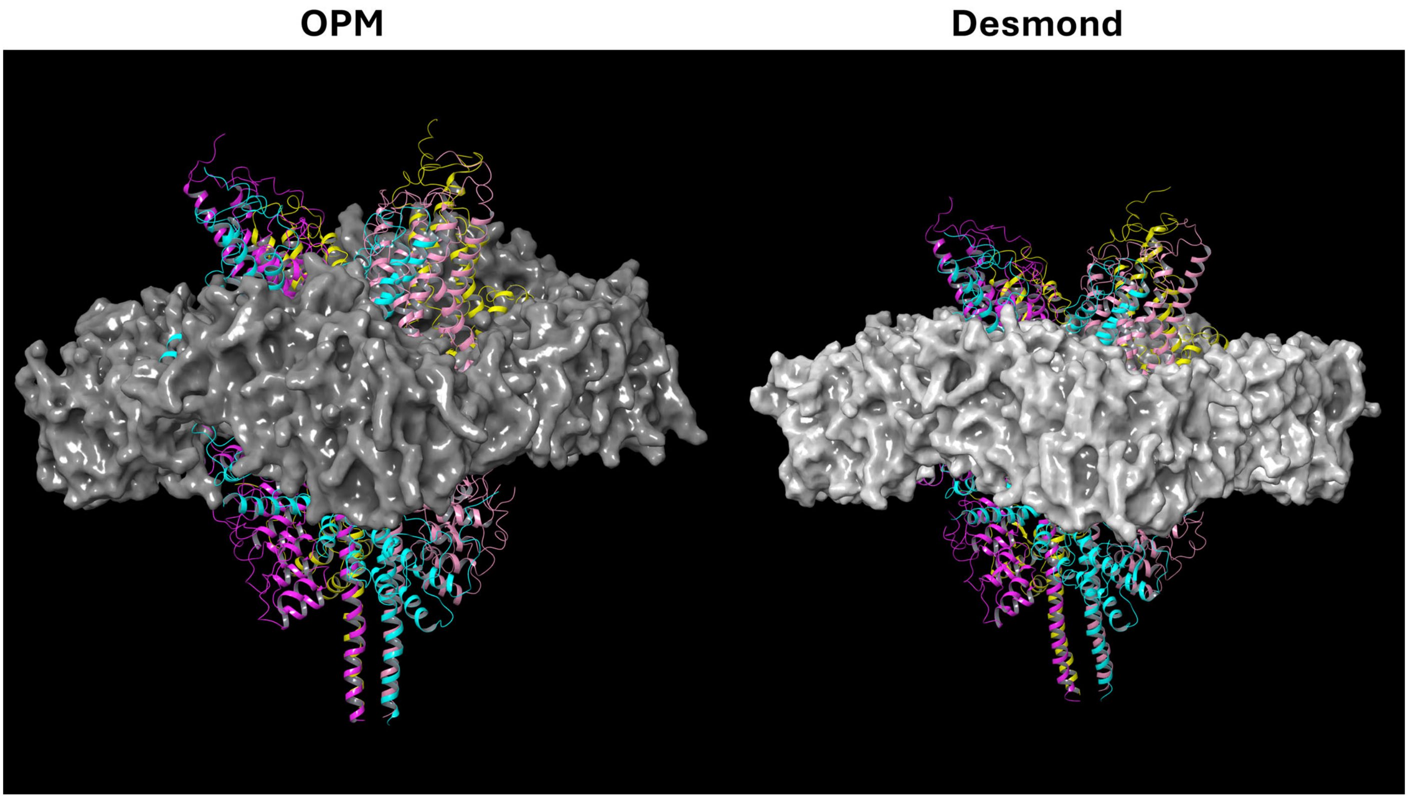

Figure S5. Comparison of the membrane–TRPC6 system after the initial relaxation. It can be observed that the system assembled by Maestro's System Builder (right) achieves a more uniform distribution of phospholipids compared to the initial system based on OPM coordinates (left).

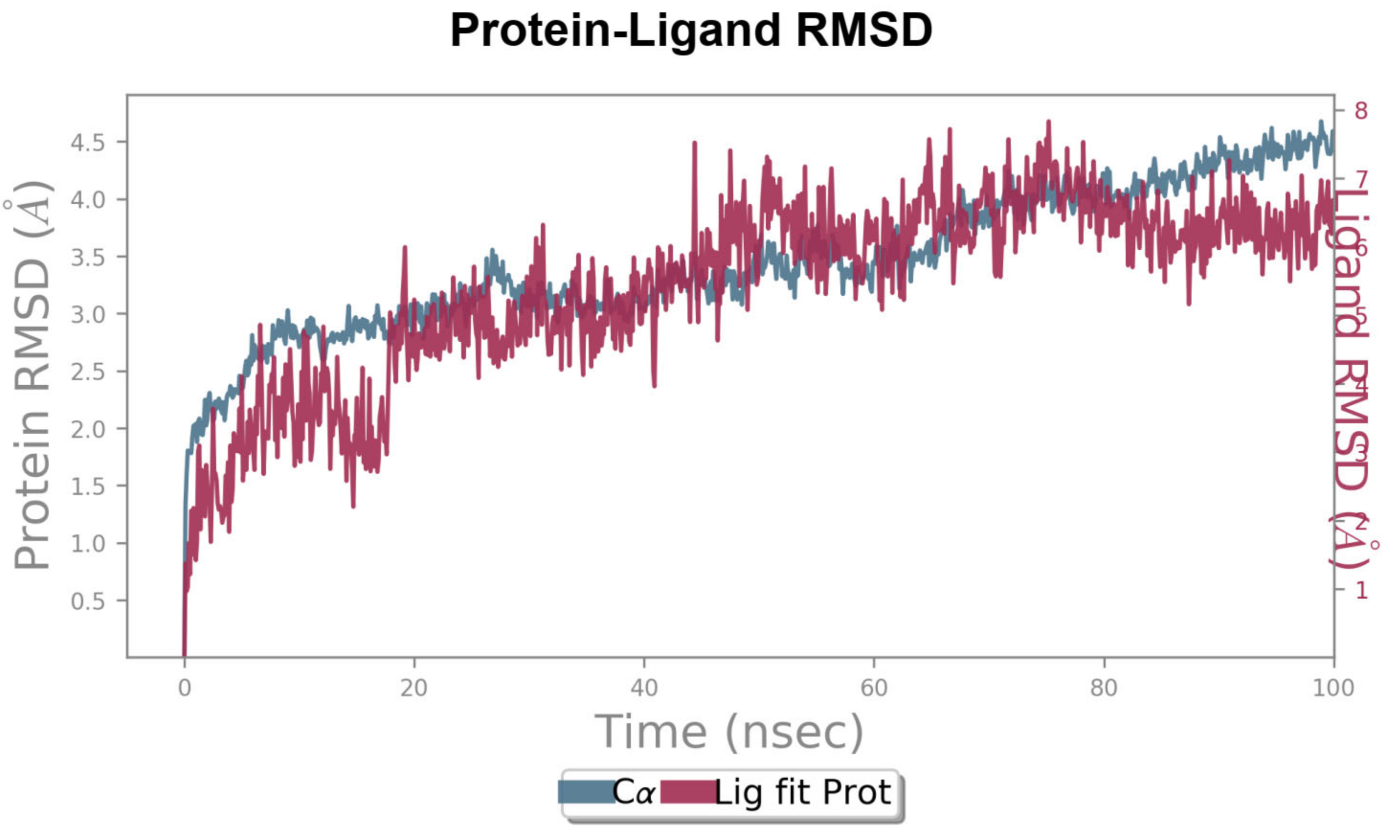

Figure S6. RMSD plot for the BT11–TRPC6 complex based on the system built using the membrane coordinates from OPM. The blue line represents the RMSD of the protein, and the red line represents that of the complex. The protein RMSD never reached equilibrium throughout the simulation time. Based on this result, we propose that the default model (Desmond's System Builder) is superior to the one provided by OPM for 100-ns simulations.
